# Multiple Effects of Ascorbic Acid against Chronic Diseases: Updated Evidence from Preclinical and Clinical Studies

**DOI:** 10.3390/antiox9121182

**Published:** 2020-11-26

**Authors:** Massimiliano Berretta, Vincenzo Quagliariello, Nicola Maurea, Raffaele Di Francia, Saman Sharifi, Gaetano Facchini, Luca Rinaldi, Michela Piezzo, Ceccarelli Manuela, Giuseppe Nunnari, Monica Montopoli

**Affiliations:** 1 Department of Clinical and Experimental Medicine, University of Messina, 98121 Messina, Italy; giuseppe.nunnari@unime.it; 2 Division of Cardiology, Istituto Nazionale Tumori—IRCCS Fondazione “G. Pascale”, 80131 Napoli, Italy; quagliariello.enzo@gmail.com (V.Q.); n.maurea@istitutotumori.na.it (N.M.); 3 Italian Association of Pharmacogenomics and Molecular Diagnostics (IAPharmagen), 60126 Ancona, Italy; rdifrancia@iapharmagen.org; 4 Department of Pharmaceutical and Pharmacological Sciences, University of Padova, 35100 Padova, Italy; saman.sharifi@phd.unipd.it (S.S.); monica.montopoli@unipd.it (M.M.); 5 Division of Medical Oncology, “S. Maria delle Grazie” Hospital—ASL Napoli 2 Nord, 80126 Pozzuoli, Italy; gafacchi@libero.it; 6 Department of Advanced Medical and Surgical Sciences, University of Campania “L. Vanvitelli”, 80121 Napoli, Italy; lucarinaldi@hotmail.it; 7 Division of Breast Medical Oncology, Istituto Nazionale Tumori—IRCCS Fondazione “G. Pascale”, 80131 Napoli, Italy; m.piezzo@breastunit.org; 8 Division of Infectious Disease, University of Catania, 95122 Catania, Italy; manuela.ceccareli86@gmail.com

**Keywords:** ASC, antioxidant, cancer, immune system, cardiovascular diseases, infectious diseases

## Abstract

Severe disease commonly manifests as a systemic inflammatory process. Inflammation is associated withthe enhanced production of reactive oxygen and nitrogen species and with a marked reduction in the plasma concentrations of protective antioxidant molecules. This imbalance gives rise to oxidative stress, which is greater in patients with more severe conditions such as sepsis, cancer, cardiovascular disease, acute respiratory distress syndrome, and burns. In these patients, oxidative stress can trigger cell, tissue, and organ damage, thus increasing morbidity and mortality. Ascorbic acid (ASC) is a key nutrient thatserves as an antioxidant and a cofactor for numerous enzymatic reactions. However, humans, unlike most mammals, are unable to synthesize it. Consequently, ASC must be obtained through dietary sources, especially fresh fruit and vegetables. The value of administering exogenous micronutrients, to reestablish antioxidant concentrations in patients with severe disease, has been recognized for decades. Despite the suggestion that ASC supplementation may reduce oxidative stress and prevent several chronic conditions, few large, randomized clinical trials have tested it in patients with severe illness. This article reviews the recent literature on the pharmacological profile of ASC and the role of its supplementation in critically ill patients.

## 1. Introduction

Vitamin C, also known as ascorbic acid (ASC), is a key antioxidant, a cofactor in essential enzyme reactions, and a key nutrient.

In several animals, it is synthesized in the liver or kidneys, whereas some species, such as humans and non-human primates, have lost this ability due to mutations in the coding sequence of the last committed enzyme of the pathway and must obtain it from the diet [1]. The form of ASC found in dietary sources is l-threo-hex-2-enono-1,4-lactone, which was originally called hexuronic acid. When, in the early 1930s, Szent-Györgyi identified and isolated it as the molecule capable of preventing and treating scurvy, it was renamed ascorbate. Scurvy typically developed during long sea voyages, weeks after the provisions of fresh fruit and vegetables had run out [2]. Failure to treat it led to death. Seamen discovered that it could be treated and prevented by citrus fruit, even though this lay remedy was scorned by physicians as well as scientists.

The enzymatic reactions for which it is a cofactor involve dioxygenases, which participate in a wide range of physiological processes including the synthesis of collagen, carnitine, norepinephrine, and serotonin;the regulation of hypoxia-inducible transcription factor (HIF); and histone demethylation [3,4].

ASC is an essential component of the human diet, found in a wide range of food products, especially fresh vegetables and fruit [5]. In adults, the recommended daily dose of around 100 mg has been demonstrated to ensure a plasma concentration of 50 M. Since ASC performs most of its functions inside cells, it is transported through their plasma membranes. ASC exists in two molecular forms thatare characterized by different chemical stabilities, half-lives in vivo, and transport mechanisms. The oxidized form, dehydroascorbic acid (DHA), is transported from the extracellular medium into the cell by glucose transporters (GLUTs), whereas the reduced form, ASC, is transported by sodium–ASC co-transporters (SVCTs) [6,7].

ASC also plays a well-documented therapeutic role in conditions such as cancer, cardiovascular disease (CVD), and infectious disorders; however, rigorous clinical trials are too few to allow drawing any conclusions, especially where cancer is concerned.

Here, we aimed to summarize the main biological effects of ASC, and its effects on cancer survival and cardiovascular and infectious diseases, as well as the clinical trials currently available in the literature; considering the lack of knowledge on the interactions between drugs and ASC as well as on the putative cardioprotective properties of ASC supplementation, we highlight the beneficial or harmful effects of ASC, identifying the state-of-the-art knowledge on these topics.

## 2. Materials and Methods

A systematic search of the Medline and EMBASE databases was performed to identify potentially relevant papers reporting original research on the activity of ASC in humans. Preclinical and/or clinical studies of the role of ASC, published in English with available abstracts, were selected if they addressed one or more of the following topics: immune system homeostasis, cancer, CVD, infectious diseases, and pharmacological processes, including pharmacokinetics and the risk of interactions. Reviews, case reports, and studies lacking immunomodulation activity endpoints or conducted in surgical settings were excluded.

The following search strings were used: “ASC OR Ascorbic acid AND cancer”, “ASC OR Ascorbic acid AND infection”, “ASC OR Ascorbic acid AND cardiovascular”, “ASC OR Ascorbic acid AND immunity system”. The databases were last accessed on 31 May 2020.

Independent searches were performed by four of the authors (B.M., R.D.F., Q.V., and M.M.), who selected the eligible papers. Any disagreements were resolved by consultation and discussion with an assessor (G.F.).

A Preferred Reporting Items for Systematic Reviews and Meta-Analyses (PRISMA) flow diagram (http://prisma-statement.org/PRISMAStatement/FlowDiagram.aspx. Downloaded 10 June 2020) was prepared to illustrate the systematic review process.

**Figure 1:**
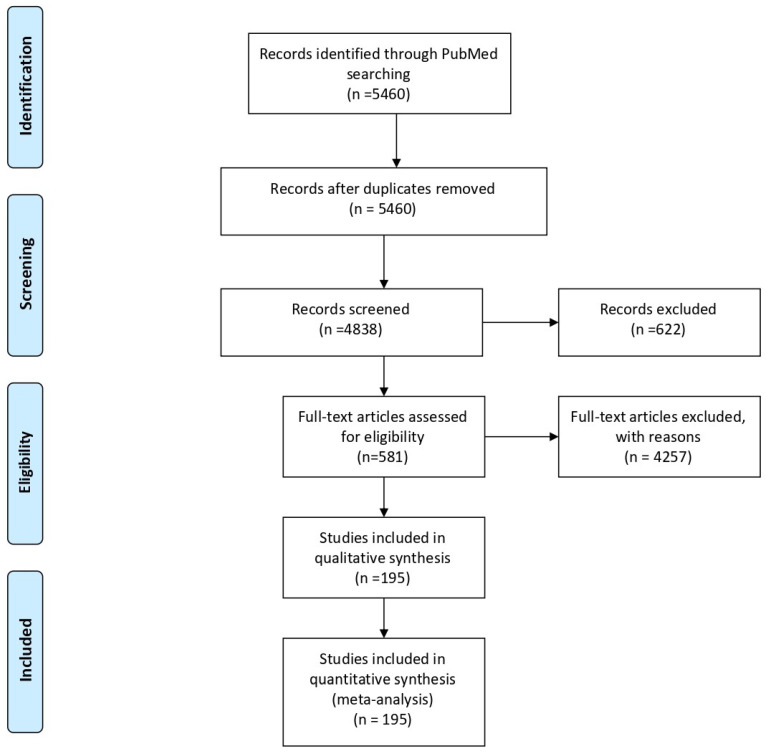
Flowchart of the identified, screened and included in this review.

The systematic review was summarized according to PRISMA (Preferred Reporting Items for Systematic Reviews and Meta-Analyses) guidelines and represented in Figure 1.

## 3. ASC Pharmacokinetics

The pharmacokinetics of orally administered ASC are non-linear and differ widely from thoseof most low-molecular-weight drugs [8]. However, scanty information hampers the interpretation of the results of clinical studies [8,9,10]. The pharmacokinetics of ASC aredetailed below.

### 3.1. ASC Absorption

**Figure 2:**
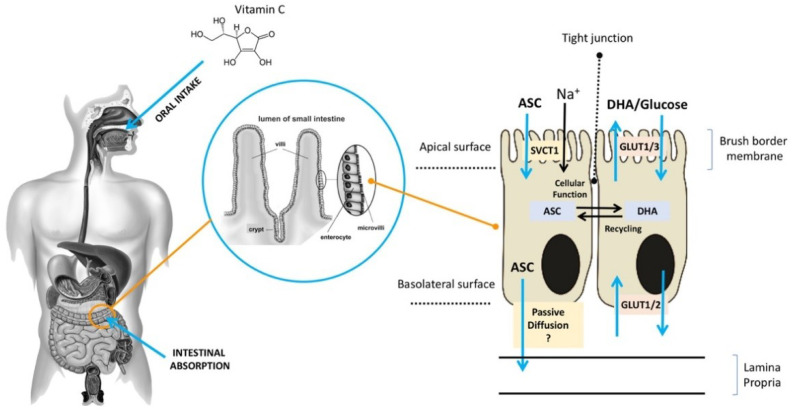
Absorption of ASC across the brush border membrane in intestinal epithelial cells (ASC: ascorbic acid;DHA: dehydroascorbic acid;SVCT: sodium–ASC co-transporters;GLUT: glucose transporters).

ASC is mainly obtained from dietary sources, especially vegetables and fruit [11]. Diets rich in ASC provide an adequate amount of the vitamin for healthy individuals [12], but they may be insufficient for those who suffer from chronic ASC insufficiency due to lifestyle habits (smokers) or to disease (scurvy) [8,13,14]. ASC is found in a reduced (ASC) and an oxidized (DHA) form [15]. It is highly water-soluble, and more than 99.9% is available in an anionic form at a pH of 7.0, which may slow down its diffusion rate across the plasma membrane even in the presence of a significant concentration gradient. In acidic environments, ASC is commonly found in an un-ionized form, which facilitates its absorption, largely through passive diffusion. After the oral administration of ASC, individuals with normal levels of ASC have similar times to maximal plasma concentration, although it is unclear whether passive diffusion contributes significantly to its absorption through this route [16,17] (Figure 2). Active transport plays a key role in ASC absorption independently of the concentration gradient. As early as the 1970s, ASC bioavailability was reported to be highly dose-dependent [18,19,20,21]. Increasing oral doses were found to be associated with decreasing absorption rates, and several studies reported that intestinal ASC absorption is a function of saturable active transport [22,23]. As shown in Figure 1, after oral intake, ASC is mostly absorbed by brush border membrane cells of the intestinal epithelium. In the gut, ASC and DHA are taken up by distinct mechanisms [24,25]. ASC absorption is sodium-dependent and is mediated by SVCT1, a sodium-coupled active transporter [16,18]. The SVCT family was discovered by Tsukaguchi and co-workers, who identified the low-affinity/high-capacity active transporter SVCT1 in the intestine [7]. SVCT1 is also expressed in the epithelium of the proximal renal tubule, where it is responsible for active ASC resorption in the kidney [16]. Indeed, in Slc23a1−/− mice, which lack SVCT1, the renal fractional excretion of ASC is increased by up to 18 times, whereas its intestinal absorption is not significantly reduced [25]. These findings support the notion that renal SVCT1-mediated resorption is crucial for ASC homeostasis. The action of SVCT1 is dose-dependent, and in several tissues, its expression seems to be modulated by ASC concentrations [26]. Although the mechanisms responsible for ASC efflux to plasma are still unclear, volume-sensitive anion channels are conceivably involved [19]. As regards DHA, its absorption is mediated by GLUT1 or GLUT3; as a consequence, DHA competes with glucose for transport, and its uptake is hampered by excess glucose; nonetheless, in the absence of glucose, ASC and DHA show similar maximal uptakes [18]. DHA transport to the circulation through the basolateral membrane and its transformation to ASC in cells may contribute to DHA uptake by keeping the intracellular concentrations of ASC at a low level [20,21]. In sum, ASC is easily carried across the apical membrane of intestinal epithelial cells by active transport, whereas the mechanisms involved in its efflux to the circulation are still unclear. Although intracellular ASC is efficiently kept reduced, thus facilitating further DHA uptake, DHA efflux to the circulation through the GLUTs does not play a significant role. Since the neutral intracellular pH involves a predominance of the anionic form (99.9%), the hydrophilicity of ASC results in fairly slow passive efflux [27]. These findings suggest the presence of yet-unknown transporters/channels that facilitate ASC efflux.

### 3.2. ASC Distribution

The distribution of ASC is highly compartmentalized. Notably, increasing intake does not raise steady-state plasma concentrations beyond 70–80 µM [17]. A daily intake of 200–400 mg results in plasma saturation [28]. In some individuals (e.g., smokers) and conditions (e.g., pregnancy and a variety of diseases), a higher intake is required to maintain a sufficient concentration. However, its transport across membranes, at least its distribution from blood to tissues, is unlikely to be mainly achieved through simple diffusion. In healthy individuals, intracellular ASC concentrations range from about 0.5 to 10 mM, compared to a mere 50–80 μM in the plasma [29]. The low plasma concentrations of oxidized ASC (DHA) found in healthy subjects exclude GLUT-mediated transport and play a key role in ASC distribution. The molecular mechanisms underpinning the widely different steady-state concentrations of the vitamin found in different tissues are largely unknown. Until additional tissue-specific SVCT2 isoforms are identified, the steady-state concentrations of ASC in the various organs may be ascribed to SVCT2 levels in cells and to ASC plasma concentrations.

### 3.3. ASC Metabolism

The metabolism of ASC is closely associated withits redox status. ASC is a chain-breaking antioxidant that quenches free radicals and donates electrons to a large number of mono- and dioxygenases [30,31]. Moreover, a variety of mechanisms ensure that most oxidized ASC is recovered by intracellular recycling and converted back to ASC, its biologically active reduced form, by several cell types [32]. Since in humans, the daily ASC turnover is only 3% [33], the close regulation of its daily intake is essential to maintain a sufficient level. DHA conversion to ASC correlates with dose-dependent renal reuptake and is involved in preserving ASC homeostasis in the body [33,34,35,36,37,38].

### 3.4. ASC Excretion and Resorption

Due to its low molecular weight and high hydrophilicity, ASC is efficiently excreted through the kidneys. It is mostly filtered through the glomerulus by virtue of the hydrostatic pressure gradient and is concentrated in pre-urine after water resorption. Its increase from <0.01% in plasma to about 15% in pre-urine represents a concentration gradient of 1500:1. Reuptake in the proximal renal tubules is mediated by saturable active transport via SVCT1. The excretion of excess ASC in individuals with plasma saturation can be quantified [17]. In subjects with ASC deficiency, resorption is mainly performed by SVCT1 in the apical membrane, although diffusion from the luminal surface may also contribute to the overall uptake. The renal reuptake of ASC is highly concentration-dependent. Its renal excretion coefficient ranges from 0 to 1, depending on the individual’s ASC concentration; this confirms its predominant reuptake in individuals with ASC deficiency and its predominant excretion in those with saturation [17,35]. In healthy individuals with a daily intake >500 mg, the value of the coefficient is 1, suggesting a minor role for passive renal resorption [17,35].

## 4. Mechanism of Action of Ascorbic Acid

**Figure 3:**
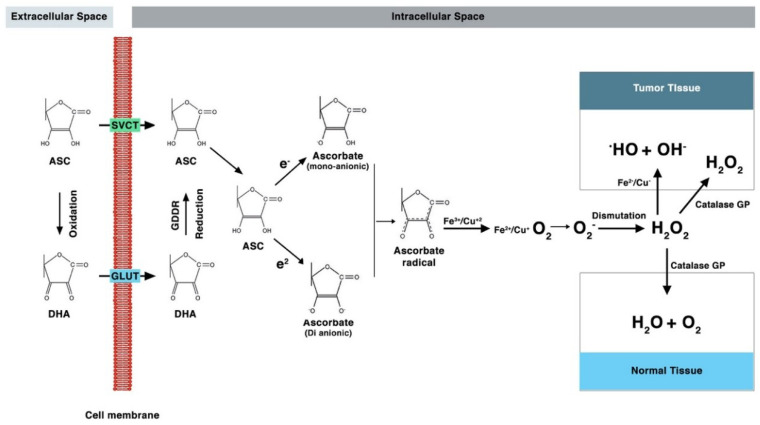
Mechanism of action of Vitamin C, and its oxidized forms’ cellular uptake via different transporters (ASC: ascorbic acid; DHA: dehydroascorbic acid; SVCT: sodium–ASC co-transporters; GLUT: glucose transporters; GDDR: glutathione-dependent dehydro-ascorbate reductase).

In humans, exogenous ASC is a cofactor in the formation of 4-hydroxyproline in –XASC–Pro–Gly– sequences in collagens and in several other proteins and is thus essential for collagen formation and tissue repair. ASC is reversibly oxidized to DHA in cells. In healthy individuals, more than 99% is found in an anionic form. The sequential donation of two electrons (oxidation) from the double carbon bond produces free radical ascorbate (hemi dehydroascorbic acid). Ascorbate radicals have a half-life of a few milliseconds to several minutes, depending on the presence of oxygen and metals, as shown in Figure 3 (primarily Fe^++^ and Cu^++^). Since this is a reversible reaction, in physiological conditions, ascorbate radicals can be converted back to ASC (reduction). Furthermore, the loss of the second electron (oxidation) leads to the formation of DHA, which is an important source of ASC for red blood cells. Intracellular ASC regulates several processes involved in metabolic homeostasis, such as oxygen sensing and hypoxic regulation (the inhibition of hypoxia-inducible factor), energy production (involving interactions with cytochrome P-450 and nicotinamide dehydrogenase), the regulation of amino acid oxidase, carbohydrate metabolism, the synthesis of lipid-based hormones and neurotransmitters, iron metabolism and others [29]. Iron also plays a role in host defense and inflammation by activating several enzymes such as myeloperoxidase, NADPH oxidase, indoleamine 2,3-dioxygenase, nitric oxide synthesis, and lipoxygenases [37]. Notably, ASC has been demonstrated to play a major role in key proteins that regulate gene expression [38]; in fact, in recent years, increasing interest has been attracted by its interactions with medications, the influence of individual genetic profiles on its uptake, and its epigenetic function in cancer prevention [38].

## 5. Drug Interactions

Recent advances in pharmacogenetics have allowed identifying prognostic and predictive markers that can be used to enhance the efficacy of drug treatments in individual patients. The need to avoid occult interactions between treatments and nutrients (including ASC) in cancer patients has been attracting mounting attention. The following evidence-based diagnostic table (Table 1) reports the most common drug–ASC interactions garnered from the studies included in the present review.

## 6. Genetic Variance of ASC Uptake

About 200 single nucleotide polymorphisms (SNPs) have been identified in the exonic (coding) and intronic (non-coding) loci of the genes SVCT1 (SLC23A1) and SVCT2 (A2) [40]. However, to date, no SNPs have been associated with specific defects in ASC uptake, largely because there are several different SVCT SNPs, all of which are uncommon in the general population [41]. According to numerous studies, by reducing ASC renal absorption, defects in the human SVCT1 and SVCT2 genes could play a role in recapitulating low ASC concentrations with intakes in the range of 30 to 2500 mg/day [42]. Low levels of ASC induced by SVCT1 and SVCT2 SNPs have been associated with low enzymatic activity in the lens and aqueous humor [25]. A significant correlation, described between SVCT2 SNPs and colorectal adenoma [43], has not been found in other work. Another study has reported a correlation between an SVCT1 SNP and an increased risk of developing Crohn’s disease [44]. Five studies involving about 18,000 patients have described a very low correlation between SVCT1 SNPs and the risk of developing CVD [45]. Conceivably, reduced ASC transport due to SVCT1 and SCVT2 deficits can be mitigated by dietary ASC. In conclusion, the clinical and physiological significance of SVCT SNPs is still unclear. Detailed data on role of the genetic polymorphisms involved in ASC pathophysiology are not reviewed herein [46].

## 7. Epigenetic Function of ASC

Several enzymes affect DNA methylation and chromatin changes and may induce genomic instability and increased chromosomal fragility. This epigenetic change is the most striking cause of DNA hypomethylation and a major risk factor for cancer development. Furthermore, whereas specific functions have been identified for a number of these enzymes, the regulators of many others are still unknown [47]. Establishing their mechanism of action would allow learning whether drugs and vitamin supplements have an actual impact on their biological functions. ASC has a direct epigenetic involvement in the 2-oxoglutarate-dependent dioxygenase family (2-OGDDs), where ASC serves as a cofactor in enzymatic reactions. The key enzymes of the family are Jumonj C-domain-containing histone demethylases (JMJCs) and ten-eleven translocation (TET) enzymes. In a human embryonic stem cell model, ASC induced extensive DNA demethylation; this effect was most clearly observed in genes characterized by bivalent chromatin modifications and in those that become demethylated during reprogramming [48].

The gene complexes involved in epigenetic modifications affected by ASC supplementation are summarized in Table 2.

## 8. Immune Modulation by Ascorbic Acid

The immune system consists of several organs, tissues, cells, and proteins that combat bacteria, viruses, and cancer cells [56]. ASC exerts multiple effects on the viability and metabolism of human immune cells [57]. The main natural sources of ASC are summarized in Table 3 [58].

Severe ASC deficiency leads to scurvy [59], a disease characterized by the disturbance of collagen metabolism (resulting in poor wound healing) and by impaired immunity (resulting in a high risk of pneumonia and fatal CVD) [60]. Patients are treated with 10 mg/day of ASC; notably, humans need much higher doses of ASC than of other vitamins [61,62]. In healthy subjects, a diet supplying 100–200 mg/day of ASC achieves plasma saturation and is believed to prevent the most common chronic diseases [63] by providing continuous immune cell stimulation [64]. ASC has a number of properties that contribute to its immune-modulating effects [64]. As an antioxidant—based on its ability to donate electrons to molecules—it inhibits the oxidation of proteins, lipids, carbohydrates, and nucleic acids [65] due to smoking habits [66], chemical agents, endocrine disruptors [67], drugs [68], and toxins. It is also a cofactor for mono- and dioxygenase, which stabilize collagen fibers, which in turn provide an optimal microenvironment for immune cells [69]. Moreover, ASC is a cofactor for the two hydroxylases involved in carnitine biosynthesis, which activate mitochondrial fatty acid oxidation, leading to ATP synthesis [70].

**Figure 4:**
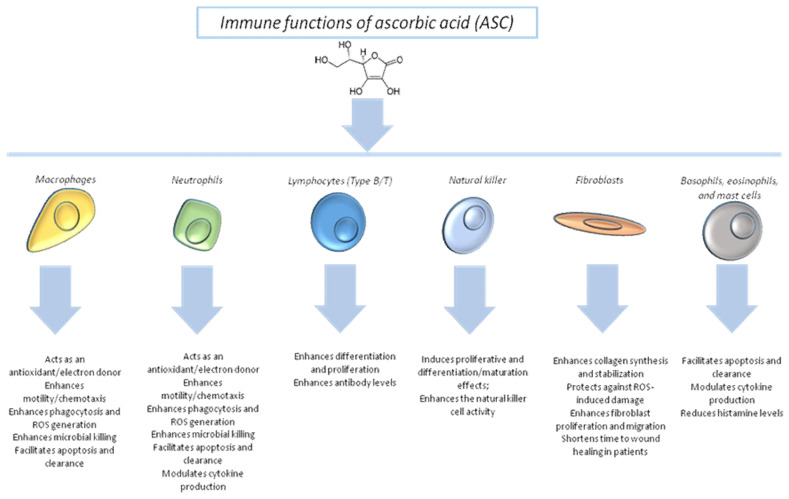
Overall clear effects of ASCon human immune cells.

ASC is stored in leukocytes [71], where it accumulates at 50- to 100-fold higher concentrations than in the plasma [72]. Maximal ASC concentrations in leukocytes are achieved with dietary intakes of ~100 mg/day [72]; neutrophils accumulate up to 1–2 mM ASC through SVCT2 [73]. These concentrations confer protective effects on immune cells. In healthy volunteers, supplementation with dietary or gram doses of ASC enhanced neutrophil chemotactic ability [74]. In patients with poor ASC intake (<50 µM), the administration of high ASC doses (250 mg/day) increased neutrophil chemotaxis [75], whereas very high ASC doses (1 g/day) combined with vitamin E significantly enhanced neutrophil function and chemotaxis [76]. Oral ASC thus improves immune cell function, particularly in individuals with inadequate ASC intake (Figure 4).

In cancer patients, ASC modulated the tissue microenvironment by reducing the levels of proinflammatory cytokines [77]. Interestingly, the reduction of interleukin (IL)-1 and IL-6 in such patients exerted benefits against both cancer recurrence and CVD [78]. To date, a single study has investigated the effect of ASC supplementation on neutrophil apoptosis in patients with sepsis [79]. The intravenous (iv) administration of 450 mg/day of ASC to patients with abdominal sepsis reduced caspase-3 levels in peripheral blood neutrophils, highlighting its antiapoptotic effects on immune cells [80]. Several studies directed at establishing the anti-inflammatory and antiapoptotic effects of ASC on neutrophils are currently under way.

The effects of ASC on lymphocytes (Figure 4) are less clear-cut, although an antioxidant action has been suggested. The incubation of lymphocytes with subclinical ASC concentrations promoted cell proliferation, enhanced antibody production, and reduced apoptosis due to exposure to chemicals and toxins [81]. Notably, ASC appears to have an important role in inducing T-cell differentiation and maturation (Figure 4), i.e., immune cell stimulation [82]. In addition, proliferation and differentiation/maturation were enhanced in natural killer cells [83], also in cancer patients. Intraperitoneal administration enhanced humoral antibody levels and lymphocyte activity in a Guinea pig model [84]. A human intervention study has described favorable associations between IgM/G and A and ASC supplementation [85]; interestingly, ASC stimulated protein synthesis and plasma cell maturation [85]. A clinical study of patients with asthma demonstrated that iv ASC increased immune modulation and reduced the severity of asthma symptoms (Figure 4) [86]. In addition, ASC enhanced ex vivo lymphocyte proliferation in elderly individuals [85].

ASC also modulates the cytokines involved in inflammation and the immune response. The incubation of peripheral blood lymphocytes with ASC reduced the lipopolysaccharide (LPS)-induced generation of tumor necrosis factor (TNF)-α and interferon (IFN)-γ, two proinflammatory cytokines, and increased the expression of the anti-inflammatory IL-10 [87]. Monocytes isolated from pneumonia patients and treated with ASC had reduced levels of the proinflammatory cytokines TNF-α and IL-6, suggesting potential benefits in such patients [88]. An immune-stimulating action of ASC has been clearly demonstrated against viral infection, since fibroblasts incubated with Influenza A virus and ASC showed an enhanced production of IFN (which has antiviral activity) [89]. Another interesting clinical trial has found that, following stimulation with bacterial antigens (LPS), oral ASC (1 g/day) enhanced peripheral blood mononuclear cell-derived IL-1, IL-10, and TNF-α [90].

## 9. Cancer

The value of iv ASC as a complementary agent in cancer treatment has been studied since the 1970s [91,92]. More than 50 years on, it is still unclear how ASC specifically affects cancer cells. Until recently, little was also known about the physiological aspects of their interaction, i.e., how cancer cells acquire ASC and how it is metabolized or compartmentalized inside them. Based on the well-established role of ASC in collagen synthesis, in the 1950s, McCormick hypothesized that metastases could be related to poor collagen formation and connective tissue degeneration as a result of ASC deficiency, and that ensuring optimal collagen synthesis by ASC supplementation could stem the process [93,94]. However, the benefits of ASC in cancer treatment are still controversial.

In the 1970s, support for McCormick’s hypothesis came from Cameron and co-workers, who suggested that ASC inhibits the enzyme hyaluronidase, which reduces tissue disruption and cancer cell proliferation [95]. Cameron’s first clinical trials, initially with Campbell [96,97] and later with the Nobel Prize winner Linus Pauling [92], examined the anticancer effect of ASC. Two retrospective trials suggested to Cameron and Pauling that high-dose ASC had a therapeutic effect in advanced cancer, since in terminal patients, oral ASC added to the cancer treatment resulted in longer survival and symptomatic relief compared to control patients [91,92]. Their study was criticized, among other things, because of the lack of blinding, inherent in a retrospective trial, and of the possible placebo effect of ASC administration. Studies conducted at the Mayo Clinic re-evaluated these findings by analyzing the efficacy of ASC in randomized, double-blind, placebo-controlled trials. The negative findings of two subsequent prospective studies [98,99] of oral ASC forthe survival of patients with advanced cancer compared to patients receiving placebo all but stopped research in this field for several years. However, none of the above studies measured ASC plasma levels, a key issue for understanding the real effect of ASC administration on patients. The question was clarified by research conducted by the National Institute of Health (NIH) to establish ASC dietary recommendations [100,101]. Analyses performed in healthy young subjects showed that ASC’s pharmacokinetics depend on the route of administration: oral ASC achieved low plasma concentrations (100–200 µM), whereas iv ASC resulted in 100-fold higher concentrations (around 15 mM) [102], due to the limited intestinal absorption, renal resorption, and excretion of oral ASC. Since the iv route bypasses these processes, it achieves high plasma concentrations that are safe or tolerable for humans [102]. Thus, high “pharmacological” concentrations are achieved only by intravenous, not oral, “physiological” administration. The consequent suggestion that only pharmacological concentrations may act as a drug has revived the interest in using ASC as an anticancer agent.

### 9.1. Effect of Pharmacological ASC on Cancer Cells

After the investigations of ASC’s pharmacokinetics, several studies investigated the effect of pharmacological ASC doses on cancer cells. In vitro studies conducted in a number of human and mouse cancer cell lines initially showed that concentrations around 20 mM selectively killed cancer cells but had no adverse effects on normal cell lines. In the mid-2010s, Chen and colleagues advanced the hypothesis that the mechanism inducing cancer cell death depended on hydrogen peroxide (H_2_O_2_) formation, with ASC radicals as intermediate species [103]. The same team showed that in rats, iv or parenteral pharmacological doses induced ASC radical and H_2_O_2_ formation in the extracellular medium [104]. The intraperitoneal administration of pharmacological ASC doses has also been demonstrated to reduce human ovarian cancer, mouse pancreatic cancer (PC), and rat glioblastoma growth rates through a pro-oxidant effect [105], the cytotoxicity being related to the production of extracellular H_2_O_2_ and the involvement of intracellular transition metals [105,106]. Several studies have described the induction of reactive oxygen species (ROS) by high ASC concentrations in cancer cells in human PC [107], mesothelioma [108], and breast cancer (BC) [109], among others. In vitro experiments testing ASC’s compatibility with other anticancer substances disclosed a synergistic action with some of them [110,111,112,113,114]. Espey and co-workers showed that gemcitabine combined with ASC had a synergistic cytotoxic effect on eight PC cell lines due to the pro-oxidant effect of ASC through an increase in H_2_O_2_ production [110]. In mice bearing PC xenografts, gemcitabine and ASC induced greater cancer growth inhibition than gemcitabine alone [110]. ASC also acts synergistically with two antiblastic drugs (ADs) used to treat ovarian cancer, since carboplatin and paclitaxel inhibited tumor growth in mouse models of ovarian cancer and reduced the adverse effects of ADs in patients with the same disease. In triple-negative BC (TNBC), ASC combined with auranofin, which targets thioredoxin reductase (TRXR) [111,112], induced the production of extracellular H_2_O_2_ and cytotoxic effects in a BC-derived cell line (MDA-MB-231) as well as in xenografts implanted in mice. A synergistic effect of ASC in combination with ionizing radiation has also been described in several cancers [115]. In vitro, ASC enhanced the cytotoxic effect of ionizing radiation on various PC cell lines, but not on non-tumorigenic cell lines; this synergistic effect was attributed to oxidative damage due to an increase in oxidative stress in cancer cells [115]. Murine models of PC receiving radiation therapy (RT) and ASC exhibited tumor growth reduction and increased survival without the typical gastrointestinal side effects, prompting the suggestion that ASC can be used as a complementary treatment withRT in PC patients [115]. The toxicity of ASC could also be associated with its oxidation byproducts [116]. Lu and colleagues [116] reported that DHA, which is transported by GLUTs, enhanced the efficacy of oxaliplatin through redox modulation in a gastric cancer model [116]. The question of whether ASC or DHA is the more cytotoxic species for cancer cells has recently been addressed in BC-derived cell lines. ASC showed higher toxicity than DHA to TNBC cells, increasing H_2_O_2_ both in the extracellular medium and in intracellular compartments [117]. Altogether, the data showing an anticancer activity of high ASC doses, collected in the past 15 years, support their therapeutic value. However, these results have not been clearly replicated in humans. An important reason is that whereas mice synthesize ASC, humans must consumeit in the diet. This key difference must be considered when interpreting the results of xenograft models.

### 9.2. ASC Administration in Clinical Studies

Since the early 2000s, several studies and clinical trials have analyzed the effect of iv ASC in patients with different types of cancer (Table 4). Two early reports established that high-dose iv ASC was well tolerated [118,119]; however, the first study (threepatients) described extended survival [118], whereas the second (24 patients) failed to demonstrate a beneficial effect on survival [119]. In 125 breast cancer patients, iv ASC reduced AD-related side effects such as nausea, fatigue, and dizziness [120]. Similar results have been reported in 60 patients with different types of cancer, where iv ASC improved quality of life (QoL) [121]. In addition, the administration of ASC alone improved QoL in a study involving 17 patients with different solid tumors, even though it showed no objective antitumor effect [122]. Two studies investigating the effect of iv ASC on the survival of patients with stage IV PC receiving standard AD treatment described a reduction inthe tumor mass and possible improvements in survival in 14 [123] and 9 patients [124], respectively, whereas iv ASC boosted energy and induced functional improvements in 14 chemotherapy patients with different types of cancer [125]. A randomized controlled trial involving 27 ovarian cancer patients showed that iv ASC reduced AD-associated toxicity, although it exerted minimal effects on survival [111]. In 23 patients with metastatic castration-resistant prostate cancer, iv ASC did not result in disease remission and had no anticancer effect [126], whereas a study of 13 glioblastoma patients receiving RT and of 14 non-small cell lung cancer patients receiving AD found that iv ASC extended survival [127]. Similarly, iv ASC combined with AD seemed to prolong survival in 14 PC patients [128]. A study of 73 patients with acute myeloid leukemia showed that those receiving iv ASC combined with AD experienced longercomplete remission and longer survival than those treated only with ADs [129]. A retrospective study of 613 hepatocellular carcinoma patients found that iv ASC extended disease-free survival after surgery [130]. Finally, a phase I study of 36 patients with metastatic colorectal and gastric cancer reported that high-dose ASC combined with mFOLFOX6 or FOLFIRI had potential clinical efficacy [131]. Altogether, most studies found that ASC improved QoL or reduced AD-related side effects, whereas few of them described a clear antitumor effect or extended survival, especially when ASC was administered as an adjuvant. The diverse results could be related to a variety of factors such as the dosage, number of patients (often few), types of cancer, methods, and parameters studied, as stressed by several reviews [132,133,134,135]. Crucially, the factors involved in ASC transport and compartmentalization should also be considered when interpreting the clinical results and assessing the role of ASC in cancer patients (Table 4).

## 10. Role of ASC in the Management of Infectious Diseases

In infectious diseases, ASC has been investigated for its antioxidant properties. Its ability to affect neutrophil migration and apoptosis and to stimulate phagocytosis and ROS production also support a role for it as an immunomodulatory agent [136]. Several studies (Table 5) have demonstrated that ASC is capable of stimulating T cell and natural killer cell development and maturation [137,138]. Notably, patients with an infection suffer from ASC depletion that is proportional to disease severity [139]. Therefore, it is not surprising that ASC seems to exert a protective role in acute and chronic viral infectious diseases [140], which are characterized by the excess production of free radicals and DNA damage due to ROS. Chronic infectious diseases, such as human immunodeficiency virus (HIV), hepatitis C virus (HCV), and hepatitis B virus (HBV) infections, are characterized by immune dysregulation and impaired cellular metabolism, which induce a progressively worsening oxidative stress [141]. In HIV patients, oxidative stress is involved in viral replication, the inflammatory response, and reduced immune cell proliferation [141]. Moreover, HIV seems to directly affect the antioxidant defense system [142], and HIV-related oxidative stress is a concurrent cause of HIV-associated malignancies, metabolic disorders, and premature aging [142,143,144,145,146,147,148,149,150,151,152,153,154,155,156,157,158,159,160,161]. Since HIV patients have reduced levels of ASC and other micronutrients than healthy subjects [142,143,144,145,146,147,148,149,150,151,152,153,154,155,156,157,158,159,160,161,162], it has been suggested that ASC supplementation could help to reduce oxidative stress and slow down chronic inflammation in HIV-positive individuals [163]. However, high-dose ASC is capable of inducing some hepatic cytochromes, such as CYP3A, which can interact with antiretroviral drugs [141,164]. Since these interactions would cancel the positive effects of ASC, the use of dietary supplements by such patients should be supervised by the attending physicians [142]. Oxidative stress also has an important role in the development of hepatocellular carcinoma in HCV- and HBV-infected subjects [138,165]. A number of studies conducted before the direct acting antiviral (DASC) era showed that ASC supplementation improved the effects of IFN-α combined with ribavirin on alanine transferase levels [138,166,167]. Moreover, since this drug combination induced an increase in serum malondialdehyde and a concomitant reduction inglutathione peroxidase and superoxidase dismutase activities, thus increasing oxidative stress, ASC helped to prevent the excess oxidative damage and malignant cell transformation [129,168]. Indeed, Loguercio and colleagues [169] demonstrated that HCV patients with p53-positive liver biopsies were more likely to have a poor intake of antioxidant nutrients, especially ASC. Moreover, although the American Cancer Society has recommended that patients do not use dietary supplements, multivitamins can reduce the risk of cancer [170]. In the DASC era, Gonçalves and co-workers [167] reported improved antioxidant capacity, lipid profiles, and liver inflammatory markers in several HCV-positive subjects who regularly consumed orange juice during antiviral therapy (Table 5).

As regards acute viral infectious diseases, ASC has long been known to prevent and strengthen the response to acute viral infections ranging from common colds to more severe illnesses [171]; in particular, it may exert protection against sepsis-induced acute respiratory distress syndrome and viral pneumonia [140,172]. ASC is also being tested for the treatment of COVID-19 infection. In China, a phase II clinical trial ending in September 2020 has investigated whether high-dose ASC helps to improve the outcomes of patients with severe COVID-19 pneumonia treated in intensive care units, since reduced serum ASC has been reported in patients with the severe syndrome [136,137,173] (Table 5).

## 11. Cardioprotective and Vasculoprotective Properties of ASC

Too few clinical studies are available to demonstrate the beneficial effects of ASC on CVD [174] (Table 6). Whereas some studies have demonstrated favorable effects on heart failure and atherosclerosis, others have found no significant effects [175]. Information on the cardiovascular protection conferred by ASC, its effects on cardiomyocytes, its cellular uptake, and its molecular interactions is scanty. According to a meta-analysis published in 2014, oral ASC reduces blood pressure and increases endothelial function in humans, but its effects on CVD are not significant [176]; by contrast, an observational study has found that low ASC plasma levels increase the risk of CVD in men and women [177]. Interestingly, long-term ASC intake induced beneficial effects on vascular endothelial function in diabetic children and adolescents [178]. Another study has investigated the pleiotropic effects of ASC on cardiovascular health parameters including blood pressure, lipid profiles, arterial stiffness, and endothelial function [179]. According to a meta-analysis of randomized controlled trials, published in 2015, the oral administration of several natural antioxidant molecules including ASC reduced arterial stiffness and enhanced endothelial function [180]. It has also been demonstrated that supplementation with ASC and tocopherols improved endothelial function, although these effects did not extend to cholesterol or triglyceride metabolism [181] (Table 6). Notably, there are no studies demonstrating that ASC reduces total cholesterol in humans [182]. The effects of ASC on endothelial function are related to the attenuation of the oxidant effects of iron and oxygen on cells and to the regulation of mitochondrial metabolism. Two meta-analyses have demonstrated that the daily administration of 500 mg of ASC reduced systolic and diastolic blood pressure [183,184]. However, the reduction in lipid peroxidation in cardiomyocytes, induced by ASC, led to significant malondialdehyde and 4-hydroxy-nonenal inhibition [185]. In CVD patients, these mediators are associated with a reduced left ventricular ejection fraction and global longitudinal strain [186,187]. Lipid peroxidation products also play a role in cell apoptosis and necrosis and in increased levels of the cytokines involved in the cardiotoxicity of several chemicals, such as anticancer drugs [187]. ASC prevents the cardiotoxic events of lipid peroxidation, reducing the relative risk of cardiovascular events [188,189,190,191,192].

## 12. Conclusions

ASC is involved in multiple physiological processes and plays a therapeutic role in a variety of human diseases including cancer, infections, and CVD. Its activity at physiological levels is complex, dose-dependent, and compartmentalized, whereas at pharmacological levels, it is independent of dose and first-order kinetics. The insufficient understanding of its several roles has generated design imperfections, misconceptions, misinterpretations, and inaccurate conclusions. We have reviewed the most recent studies to promote a more exhaustive assessment of its role in human health and of its possible value in the prevention and treatment of chronic proinflammatory diseases. Unfortunately, the lack of accurate clinical trials prevents drawing clear conclusions on its therapeutic role; this is particularly true of highly vulnerable subjects such as long-term cancer survivors, who are at significantly greater medium- and long-term risks of one or more CVDs including heart failure/cardiomyopathies, arrhythmias, myocarditis/pericarditis, strokes, and venous thromboembolisms [193]. Since in these patients, the pathogenesis of the cardiovascular side effects of chemotherapy and targeted treatments is at least partially driven by inflammatory processes, ASC could provide a valuable preventive strategy and clearly deserves accurate testing in well-designed trials.

## Figures and Tables

**Table 1:** Some of the most common drug interactions with ASC [39].

Drug	Interactions
Amphetamine	Combination with ASC can reduce serum 2,5-dimethoxy-4-ethylamphetamine and 4-bromo-2,5-dimethoxyamphetamine concentrations.
Aluminum hydroxide (anti-Anti-glutamic acid decarboxylase remedies)	ASC can enhance aluminum hydroxide absorption, resulting in increased serum concentrations and potentially increasing the severity of adverse effects.
Bleomycin	Combination with ASC can reduce drug efficacy.
Bortezomib	Combination with ASC can reduce drug efficacy.
Chlorpropamide	ASC can reduce the chlorpropamide excretion rate, potentially raising its serum levels.
Conjugated estrogens	They can reduce ASC concentrations.
Cyclosporine	Combination with ASC can reduce drug serum concentrations.
Deferoxamine	Combination with ASC can increase the risk/severity of cardiovascular impairment.
Dienestrol	It can reduce ASC serum levels.
Diethylpropion	Combination with ASC can reduce drug serum concentrations.
Diethylstilbestrol	It can reduce ASC serum levels.
Doxycycline	Combination with ASC can reduce drug efficacy.
Erythromycin	Combination with ASC can reduce drug efficacy.
Esterified estrogens	It can reduce ASC serum levels.
Estradiol	It can reduce ASC serum levels.
Estradiol acetate	It can reduce ASC serum levels.
Estradiol benzoate	It can reduce ASC serum levels.
Estriol	It can reduce ASC serum levels.
Estrone	It can reduce ASC serum levels.
Ethinylestradiol	It can reduce ASC serum levels.
Gepefrine	Combination with ASC can reduce drug serum concentrations.
Iofetamine I-123	It can reduce ASC serum levels.
Kanamycin	Combination with ASC can reduce drug efficacy.
Lincomycin	Combination with ASC can reduce drug efficacy.
Methamphetamine	It can reduce ASC serum levels.
Promestriene	It can reduce ASC serum levels.
Quinestrol	It can reduce ASC serum levels.
Streptomycin	Combination with ASC can reduce drug efficacy.
Synthetic conjugated estrogens, A	It can reduce ASC serum levels.
Synthetic conjugated estrogens, B	It can reduce ASC serum levels.
Tibolone	It can reduce ASC serum levels.
Zeranol	It can reduce ASC serum levels.

**Table 2:** Main ASC-dependent genes and enzymes involved in epigenetic modifications [49,50,51,52,53,54,55].

Member Genes	Gene(s)	Epigenetic Functions	Phenotype Affected Functions
JMJCs, Jumonj C-domain-containing histone demethylases	Histone demethylase (HDAM)	Fe(II) 2-oxoglutarate-dependent ASC	Loss of H3 and H4 demethylation. Influence on oncosuppressor gene transcription and gene replication. Abnormal spermatogenesis [49]. Embryonic cardiomyopathy [50,51].
TET, ten-eleven translocation	TET1, TET2, TET3	Conversion of 5-methyl-cytosine (5mC) to 5-hydroxymethyl-cytosine (5hmC)	TET1 and TET2 reduction in 5hmC is implicated in the development of several types of lymphoid and myeloid acute leukemias. TET2 mutations are also associated with myeloid leukemias and myeloproliferative disorders [52].
ABH, alkylation repair homolog	H2A demethylase	Fe(II) 2-oxoglutarate-dependent ASC	H2A selective demethylase enzyme. Placental defects [52].
*FTO*, fat mass and obesity-associated gene	Methylase	Addition of 3 methyl groups to single-stranded DNA	Reduced expression due to the *FTO* rs9939609 polymorphism is associated with child obesity [49,53].
Dio3 deiodinase, iodothyronine type IIIDlk1 delta-like 1 homolog	Dio3–Dlk1 gene cluster	H3 acetylation and Lys4 demethylation	Embryonic stem cell development. Reprogramming in the absence of ASC induces hypermethylation of the imprinted Dlk1–Dio3 locus [54,55].

**Table 3:** Ascorbic acid content in fruit and vegetables (modified from [58]).

Source	Ascorbic Acid Content (mg/100 g of Product)
Acerola	1300
Apple	2–10
Apricot	7–10
Avocado	15–20
Banana	10–30
Blackberry	15
Broccoli	113
Broccoli (cooked)	90
Brussels sprouts	87–109
Cabbage (raw)	46–47
Cauliflower	64–78
Cauliflower(cooked)	55
Carrot	6
Cranberry	12
Cherry	5–8
Blackcurrant	200–210
Redcurrant	40
Damson	3
Gooseberry	40
Gourd	8
Passion fruit	25
Grapefruit	40
Guava	230–300
Horseradish	120
Kale	186
Kale (cooked)	62
Kiwi	60
Lemon	50
Lettuce	15
Lime	25
Loganberry	30
Lychee	45
Melon	10–35
Orange	50
Orange (juice)	50
Tangerine	30
Peach	7–31
Peach (canned)	6
Pepper (green)	128
Plum	3
Pea	25
Pear	3–4
Pineapple	12–25
Pineapple (canned)	12
Pomegranate	6
Potato (new)	30
Potato (Oct., Nov.)	20
Potato (Dec.)	15
Potato (Jan., Feb.)	10
Potato (Mar.–May)	8
Potato (boiled)	16
Quince	15
Raspberry	25
Rosehip	1000
Spinach	51
Spinach (cooked)	28
Strawberry	59–60
Tomato	20–25
Tomato (juice)	16
Watercress	68–79

**Table 4:** Randomized clinical studies in cancer patients treated with ASC.

Study	Type of Cancer	Administration	Outcomes
Padayatty, S. [110]	Advanced cancers	Intravenous administration	Increased survival
Hoffer, L et al. [119]	Advanced cancer or hematologic malignancy	Intravenous administration	Adverse events and toxicity were minimal
Vollbracht, C. et al. [120]	125 breast cancer patients	Intravenous administration	Significant reduction incomplaints induced by the disease and chemo-/radiotherapy, in particular, nausea, loss of appetite, fatigue, depression, sleep disorders, dizziness and hemorrhagic diathesis.
Takahashi, H. et al. [121]	Advanced cancer patients	High-dose intravenous administration	Improved Quality of Life (QoL) in cancer patients
Stephenson, C. et al. [122]	Advanced cancer patients	High-dose intravenous administration	Ascorbic acid administered i.v. at 1 g/min for 4 consecutive days/week for 4 weeks produced up to 49 mM ascorbic acid in patient’s blood and was well tolerated. The recommended dose for future studies is 70–80 g/m^2^
Monti, D. et al. [123]	Metastatic pancreatic cancer	Intravenous administration	The initial safety data do not reveal increased toxicity with the addition of ascorbic acid to gemcitabine and erlotinib in pancreatic cancer patients.
Welsh, J. et al. [124]	Metastatic and node-positive pancreatic cancer	Intravenous administration	Data suggest pharmacologic ascorbate administered concurrently with gemcitabine is well-tolerated. Initial data from this small sampling suggest some efficacy.
Hoffer, L. et al. [125]	Advanced cancer patients	High-dose intravenous administration	ASC was safe and generally well tolerated. The pre- and post-chemotherapy pharmacokinetic profiles suggested that tissue uptake of ASC increases after chemotherapy, with no increase in urinary oxalic acid excretion.
Nielsen, T. et al. [126]	Castration-resistant prostate cancer patients	Intravenous administration	Treatment with ASC did not result in disease remission.
Polireddy, K. et al. [128]	Pancreatic cancer patients	High-dose intravenous administration	Treatment with ASC was safe in patients and showed the possibility to prolong patient survival. There was no interference with gemcitabine pharmacokinetics by ASC administration.
Zhao, H. et al. [129]	Patients with acute myeloid leukemia	Intravenous administration	Patients who received ASC + DCAG (decitabine with cytarabine, aclarubicin hydrochloride, and granulocyte colony-stimulating factor) regimen had a higher complete remission (CR) rate than those who received the DCAG regimen (79.92% vs. 44.11%; *p* = 0.004) after one cycle of chemotherapy. The median overall survival (OS) was better in the ASC-DCAG group compared with the DCAG group (15.3 months vs. 9.3 months, *p* = 0.039).
Lv, H. et al. [130]	Hepatocellular carcinoma	Intravenous administration	Administration of ASC improved disease-free survival (DFS) in hepatocellular carcinoma patients (adjusted HR = 0.622, 95% CI = 0.487 to 0.795, *p* < 0.001)
Wang, F. et al. [131]	Metastatic colorectal cancer or gastric cancer	Intravenous administration	The favorable safety profile and preliminary efficacy of ASC plus mFOLFOX6/FOLFIRI support further evaluation of this combination in patients with metastatic colorectal cancer or gastric cancer
Fritz, H. et al. [132]	Cancer patients	Intravenous administration	Good safety profile and potentially important antitumor activity. ASC administration may improve the quality of life and symptom severity of patients with cancer, and several cases of cancer remission have been reported.
Jacobs, C. et al. [133]	Cancer patients	Intravenous administration	No high-quality evidence to suggest that ASC supplementation in cancer patients either enhances the antitumor effects of chemotherapy or reduces its toxicity.
Nauman, G. et al. [135]	Cancer patients	Intravenous administration	An 8.75 month increase in progression-free survival (PFS) and an improved trend in overall survival (OS) in the ASC-treated arm were seen

**Table 5:** Clinical trials and meta-analysis based on the immune-stimulating effects of ASC.

Study	Type of Patients	Administration	Outcomes
Carr, A.C. [139]	Infection diseases	Not applicable (N.A)	Low levels of ASC were proportional to infection disease severity
Tan, S.H.S. et al. [140]	Acute and chronic viral infectious	Intravenous administration	Protective effects with improvement of oxidative damage
Musisi, E. et al. [142]	HIV-infected subjects	N.A	High oxidative stress in hospitalized HIV-infected adults; ASC (*p* < 0.0001) and albumin (*p* < 0.01) were lower in HIV-patients relative to controls.
Ceccarelli, M. et al. [143]	Advanced cancer patients	High-dose intravenous administration	Improved QoL in cancer patients
Ceccarelli, M. et al. [144]	Advanced cancer patients	High-dose intravenous administration	Ascorbic acid administered i.v. at 1 g/min for 4 consecutive days/week for 4 weeks produced up to 49 mM ascorbic acid in patient’s blood and was well tolerated. The recommended dose for future studies is 70–80 g/m^2^
Ceccarelli, M. et al. [145]	Metastatic pancreatic cancer	Intravenous administration	The initial safety data do not reveal increased toxicity with the addition of ascorbic acid to gemcitabine and erlotinib in pancreatic cancer patients.
Ceccarelli, M. et al. [146]	Metastatic and node-positive pancreatic cancer	Intravenous administration	Data suggest pharmacologic ascorbate administered concurrently with gemcitabine is well-tolerated. Initial data from this small sampling suggest some efficacy.
Makinde, O. et al. [163]	Patients with HIV	Oral administration	Exogenous antioxidant supplementation with ACS did not increase the antioxidant status in patients with HIV
Madill, J. et al. [164]	Patients with hepatitis virus C	N.A	Oxidative stress was independently associated with recurrence of hepatitis virus C infection.
Gonçalves, D. et al. [167]	Patients with hepatitis C under antiviral therapy	Oral administration of orange juice	The serum levels of total cholesterol, LDL-cholesterol, c-reacrive protein (CRP) and parameters of oxidative stress decreased in patients receiving orange juice. Moreover, alanine aminotransferase (AST) levels decreased significantly after oral administration of orange juice.
Adams, K.K. et al. [171]	Patients with COVID-19	Intravenous administration	Large doses of ASC (10,000–20,000 mg/d) led to a shorter mean hospital length of stay compared with untreated patients with COVID-19 and no incidences of death

**Table 6:** Clinical trials and meta-analysis available for patients with cardiovascular diseases (CVD treated with ASC.

Study	Type of Patients	Administration	Outcomes
Ashor, A.W. et al. [176]	Patients with CVD	Oral administration	ASC reduces blood pressure and increases endothelial function in humans
Martín-Calvo, N. et al. [177]	Patients with CVD	Oral administration	Low ASC plasma levels increase the risk of CVD in men and women. ASC intake is inversely associated with cardiovascular mortality
Sabri, M. et al. [178]	Diabetic children and adolescents	Oral administration	Long-term ASC intake induced beneficial effects on vascular endothelial function, lipid profiles, and arterial stiffness
Martín-Calvo, N. et al. [180]	Patients with CVD	Oral administration	Reduction in arterial stiffness and enhanced endothelial function
Wilkinson, I.B. et al. [181]	Patients with CVD	Oral administration	Oral ASC reduces arterial stiffness and platelet aggregation
Thosar, S.S. et al. [183]	Patients with CVD	Oral administration	ASC prevents decline in endothelial function during sitting
Siavash, M. et al. [184]	Diabetic patients	Oral administration	ASC increases HDL cholesterol and reduces systolic and diastolic blood pressure
